# Laparoscopic Nephroureterectomy: The Distal Ureteral Dilemma

**DOI:** 10.1155/2009/316807

**Published:** 2008-11-05

**Authors:** Shalom J. Srirangam, Ben van Cleynenbreugel, Hein van Poppel

**Affiliations:** ^1^Department of Urology, Royal Blackburn Hospital, Blackburn, BB2 3HH, UK; ^2^Department of Urology, University Hospital KU Leuven, Herestraat 49, 3000 Leuven, Belgium

## Abstract

Transitional cell carcinoma affecting the upper urinary tract, though uncommon, constitutes a serious urologic disease. Radical nephroureterectomy remains the treatment of choice but has undergone numerous modifications over the years. Although the standard technique has not been defined, the laparoscopic approach has gained in popularity in the last two decades. The most appropriate oncological management of the distal ureteral and bladder cuff has been a subject of much debate. The aim of the nephroureterectomy procedure is to remove the entire ipsilateral upper tract in continuity while avoiding extravesical transfer of tumor-containing urine during bladder surgery. A myriad of technical modifications have been described. In this article, we review the literature and present an overview of the options for dealing with the lower ureter during radical nephroureterectomy.

Primary urothelial carcinoma of the upper urinary tract accounts for about 5% of all renal and urothelial malignancies. Though relatively uncommon, the incidence of upper tract transitional cell carcinoma (TCC) appears to be slowly increasing [1–3]. While alternative therapies, such as endoscopic ablation/resection and segmental ureteral resection, have been adopted, radical nephroureterectomy is considered the ideal treatment for upper tract TCC.

Le Dentu and Albarran performed the first complete open nephroureterectomy (ONU) for upper tract TCC in 1898 [4], but it was Kimball and Ferris in 1934 who established the need for complete removal of the ipsilateral renal tract on finding a high incidence of tumor in the remaining ureter after simple nephrectomy for upper tract TCC [5]. Upper tract TCC is frequently multifocal, has a higher rate of ipsilateral ureteral recurrence, is often associated with higher grade disease (42–47% grade 3; 18–48% grade 2) and, therefore, carries a poorer prognosis compared to bladder TCC [6, 7]. Thus in the presence of a normal contralateral upper tract, complete removal of the ipsilateral kidney, ureter, and bladder cuff remains the gold standard treatment for large, high-grade, or invasive TCC.

The procedure may be undertaken open or laparoscopically. The “standard” laparoscopic nephroureterectomy (LNU) technique has not been defined and continues to evolve. Variations include utilization of a pure laparoscopic technique versus a hand-assisted technique; transperitoneal versus retroperitoneal; and a myriad of approaches to deal with the lower ureter [8]. Indeed, the issue of the most appropriate oncological management of the lower ureter and bladder cuff has been the most debated and controversial aspect of this operation since McDonald et al. attempted to lessen surgical morbidity by advocating endoscopic resection of the distal ureter in 1952 [9]. Many approaches, varying in technique and results, have been described in [8]. Irrespective of the adopted technique, the challenge is to ensure adherence to principles of reproducibility of results, patient safety, and oncological outcomes. TCC is multifocal and even with a negative cystoscopy, up to a third of patients may have viable persistent tumor within the bladder. Some patients will have vesical or para-vesical recurrence due to urine spillage and the primary focus of the nephroureterectomy procedure remains to avoid extravesical transfer of tumor-containing urine during bladder surgery. This is the key feature of improvements in the surgical technique. In this paper, we aim to discuss the dilemma of the distal ureter and present an overview of the diverse modifications employed in managing the distal ureter.

## 1. Management of the Distal Ureter

The risk of tumor recurrence within the residual ureteral stump/periureteral meatal region in cases of incomplete upper tract removal is often cited as between 30 and 64% [10–12]. Total excision of the entire ureter, including the distal ureter with its intramural portion, the ipsilateral ureteral orifice and bladder cuff is mandatory and represents a distinct portion of the case, whether an open or laparoscopic approach to the kidney is used. Ideally, this is achieved by removal of an *en bloc*, “closed system” specimen following controlled occlusion of the ureteral orifice. Continuity of the specimen, though desirable, may be conceded to aid ease of extraction as long as the distal ureter is ligated and divided at tumor-free location. The key issue is to avoid extravesical urine contact and adherence to these principles will reduce risk of spillage or seeding of tumor cells.

The following techniques, along with some minor variations, have been advocated in order to accomplish distal ureteral removal:

- “pluck” technique,
- intussusception technique,
- pure laparoscopy,
- open resection.

## 2. Transurethral Resection of Ureteral Orifice (“Pluck” Technique)

This involves the transurethral disarticulation of the intramural ureter along with the ureteral orifice (UO) using a standard resectoscope loop or Collin's knife, usually prior to performing the laparoscopic nephrectomy. The UO is resected deep into extravesical fat allowing subsequent “plucking” of the entire ureter from above. Though the original intention was to decrease morbidity by avoiding a second lower abdominal incision during open surgery, this is a less compelling argument with laparoscopic surgery as specimen extraction mandates a larger incision.

Following UO resection, the patient is put in the flank position for LNU. The kidney is mobilized along with the ureter down to the level of the pelvic brim. Gentle traction on the ureter should result in the removal of the entire length of ureter down to the level of the detached distal ureter. An indwelling Foley catheter is left in the bladder for at least 7 days. The major concern related to this technique is the risk of tumor cell spillage into the retroperitoneum with subsequent seeding and local recurrence.

A number of authors have published small retrospective series employing the pluck technique and report no local disease recurrences [13–15]. Palou et al. reported no local recurrences after an average followup of 20 months in 31 patients with mainly high grade upper tract TCC [13]. In a large multicenter, five-institution study comprising 116 patients who underwent LNU, no difference in local recurrence was noted between the various techniques of distal ureteral removal at a median followup time of 25 months [14]. Geavlete et al. recently reported on 100 ONU patients from a single center, the majority of whom (72 cases) had undergone a pluck transurethral detachment with coagulation of the resected area [16]. The remainder (28 cases) were managed by ureteral stripping. No local recurrences were reported after a mean followup of 44 months.

Nevertheless, the propensity for urological malignancies, including TCC, to seed is well recognized [17]. Not surprisingly, therefore, local recurrence following a pluck nephroureterectomy has been noted on many occasions, often occurring early and with tragic consequences [18–22]. Ko et al. reported on 51 patients undergoing LNU with the distal ureter being managed by either the open (*n* = 30) or the pluck technique (*n* = 20) [23]. The transurethral pluck was restricted to TCC located in the calices, renal pelvis, and ureter proximal to the pelvic brim and produced recurrence rates similar to those in an open fashion. Of note, however, is that five patients had an unplanned incomplete ureterectomy and four had tumor recurrences (three in the form of metastatic disease).

Furthermore, utilizing the pluck technique is likely to permit continued urine extravasation from the cancer-bearing ureter [24, 25]. Additionally, due to the absence of an identifiable marker within the detached distal ureter, confirmation of complete specimen removal is not possible, raising the theoretical possibility of local recurrence in any remaining portion of ureter [24].

### 2.1. Pluck Technique Modifications

Various modifications on the pluck theme have been described in an attempt to minimize tumor spillage. Tan et al. advocated completion of the laparoscopic nephrectomy first with the clipping of the ureter to prevent distal migration, followed by transurethral Collin's knife mobilization of the distal ureter and bladder cuff [8]. Recently, a novel technique has been described involving an initial partial circumscribing of the bladder cuff with a Collin's knife with a 1 cm margin around the ipsilateral ureteral orifice [26]. A preformed PDS Endoloop *(Ethicon, Sommerville, NJ, USA)* is then passed through the cystoscope to ligate and occlude the ureteral orifice. The bladder cuff is then completely circumscribed down to perivesical fat and subsequently removed *en bloc* following kidney mobilization. The Endoloop also acts as a marker ensuring complete specimen removal. A similar procedure has been described using a transurethrally placed 5-mm laparoscopic hem-o-lok clip on the ureteral stump, as an alternative to the Endoloop, to ensure a closed system [27]. Both these studies report no pelvic tumor recurrences in the short term [26, 27].

### 2.2. Ureteral Unroofing

The ureteral *unroofing technique* has been described and popularized by the Washington University group [28]. This can only be employed in transperitoneal LNU and briefly comprises cystoscopic incision of the entire anterior length of the intramural ureter; electrocautery to the cut edges and floor of the intramural ureter; placement of a 7.5 F occlusion ureteral balloon catheter in the renal pelvis to prevent urine spillage; laparoscopic dissection of the kidney and ipsilateral ureter down to the level of the bladder and specimen detachment following placement of an Endo-GIA stapler on the bladder cuff. This technique has the theoretical advantage of minimizing urine leakage while maintaining a truly minimally invasive approach and promoting ureteral identification intraoperatively. It is contraindicated in the presence of active ureteral or bladder TCC. Other potential disadvantages include requirement of fluoroscopy, risk of injury to the contralateral ureteral orifice as the stapler is applied “blindly” and longer operating times [8]. Employment of the stapler device has produced hypothetical concerns relating to stone formation, tumor recurrence within urothelium trapped in the staple line, and the inability to visualize this area satisfactorily during subsequent surveillance cystoscopic inspections. These complications have not been reported to be a clinical problem. The same investigators compared LNU in 25 patients (using the unroofing technique) and ONU performed in 17 cases with a mean followup of 24 months and 43 months, respectively [29]. LNU took twice as long as ONU, but was associated with less pain, fewer complications, a shorter hospital stay, and quicker convalescence. Although there was no statistically significant difference in the disease-specific survival rate and proportion of bladder tumor recurrences, concerningly, there were 3 retroperitoneal recurrences in the LNU group. Whether this is due to surgical technique or the high tumor grade in these patients in unclear from the study.

### 2.3. Pluck Technique in HALNU

HALNU offers distinct advantages when considering how best to manage the distal ureter. Firstly, the requirement of a longer incision to facilitate a hand port will allow improved access to the bladder and distal ureter, offering the surgeon the option of either an extravesical approach, open transvesical cystotomy, or detaching the ureter using a transurethral technique [30]. In addition, the operator can facilitate dissection and resection by providing gentle countertraction on the distal ureter. Tumor spillage can be prevented by occlusion of the distal ureter by a clip or the surgeon's hand. Alternatives to the endoscopic management of the distal ureter during HALNU have been reported. Gonzalez et al. described a technique implementing insertion of a laparoscopic trochar, followed by introduction of a 24 F nephroscope allowing an endoscopic Collin's knife incision of the bladder cuff [31]. This is performed subsequent to dissection of the kidney and ureter, and after clips have been placed on the lower ureter. Alternatively, a similar technique may be performed without the need for a bladder port or patient repositioning [32]. Placement of the patient in a modified dorsal lithotomy position will permit introduction of a transurethral resectoscope to perform the bladder cuff incision. The oncological sequalae of this same group of patients was recently published, and none of the 49 patients had developed a pelvic recurrence after a mean followup period of 10.6 months [33]. Notably, the authors emphasized early ligation of ureter but did not routinely close the bladder. Vardi et al. reported a novel modification to this technique by inserting a flexible cystoscope per urethra and a 5 F electrode (ACMI, Norwalk, Conn, USA) to incise a circumferential 2-cm cuff of bladder around the UO using cutting and coagulating current [34]. Patient repositioning after the nephrectomy is avoided and the bladder opening is not closed. No pelvic recurrences were noted in their small group of patients after a mean followup of 31 months.

In summary, therefore, pluck techniques are contraindicated in the presence of lower ureteral tumor and widespread urinary tract carcinoma in situ. Coexistent bladder TCC should preclude the situation, where the bladder is left “open” with potential exposure of the perivesical tissues to malignant-cell laden urine. Patients with previous pelvic irradiation and active inflammatory conditions of the bladder are probably not ideal candidates for endoscopic procedures. Blind pulling of the ureter is discouraged to minimize ureteral tearing and the possibility of residual tissue. While retrospective studies have not confirmed the superiority of the open over the pluck method, the oncologically safe practice of maintaining a “closed” system is preferred and retroperitoneal exposure to potentially tumor cell-laden urine for any duration of time, in our opinion, is best avoided.

## 3. Intussusception Technique

This technique, first described by McDonald in 1953, has undergone various subsequent modifications around a central theme of ureteral ligation and removal either by stripping or intussusception [35]. Principles of this technique include initial catheterization of the ureter using either a ureteral catheter or a stone basket ligation and division of the ureter as part of the renal mobilization, securing of the distal ureter to the ureteral catheter/stone basket, transurethral incision of the bladder cuff, followed by removal of the distal ureter by gentle traction on the catheter via the urethra [8, 18]. The distal ureter intussuscepts into the bladder and can either be removed transurethrally or via a small lower midline incision and anterior cystotomy. A variety of technical devices, including sutures, vein stripper, balloon catheter, and double ligation, have been described in an attempt to improve ureteral excision of the ureter [18, 36–38].

Though its long-term safety during LNU has not been investigated, even after 5 years' followup, Clayman et al. reported no pelvic tumor recurrences in 14 patients undergoing ureteral stripping during ONU [36]. This is confirmed in a literature review by Laguna and de la Rosette, who compared the stripping and pluck techniques [18]. While there were no reports of local disease recurrence in the stripping group, this technique was associated with a 10% complication rate (including retained ureters and catheter breakage) resulting in an open conversion rate of between 9.5 and 12.5% in patients after difficult extraction.

Since the ureter is transected, it is contraindicated for ureteral tumors and primarily confined to low-grade renal pelvic tumors. Additionally, any cause for pelvic fibrosis, such as previous surgery or irradiation and retroperitoneal fibrosis, may further increase the risk of retention of ureteral remnant. Bladder mucosa is exposed to ureteral mucosa with the potential for seeding. Its main drawback is its failure to guarantee adequate excision of the intramural ureter and bladder cuff, potentially resulting in tumor recurrence, and is thus unlikely to gain universal acceptance following LNU.

## 4. Pure Laparoscopic

A completely laparoscopic approach offers distinct advantages in terms of blood loss, postoperative pain, recovery times, and equivalent short- and intermediate-term oncologic efficacy. The kidney and ureter are mobilized in the standard fashion and the distal ureter may be secured by one of 2 main techniques:

- laparoscopic extravesical stapling of the distal ureter [28, 39];
- transvesical laparoscopic detachment and ligation (Cleveland approach) [40, 41].

### 4.1. Laparoscopic Stapling

This method is usually combined with a ureteral unroofing procedure. The ureter is clipped early and dissected caudally until it diverges to merge with the detrusor muscle fibers at the ureterovesical junction (UVJ). Gentle traction on the ureter will tent up the wall of the bladder at the UVJ enabling placement of a 12-mm laparoscopic GIA tissue stapler (Endo-GIA; Auto-Suture, Norwalk, Conn, USA) or a large hem-o-lok clip. If desired, an ontable bladder fill with saline/indigo carmine solution may be performed to exclude extravasation and/or a delayed cystogram is obtained before catheter removal. A more recent trend has been to perform stapling of the bladder cuff as the initial step, followed by a transurethral resection of the ipsilateral UO till the staple line is reached [8].

Laparoscopic stapling has manifest advantages. It may help reduce operative time and facilitates a minimally invasive procedure while maintaining a “closed” urinary tract, thus preventing tumor spillage. There are, however, numerous concerns related to staple usage. Deployment of the stapler may prove awkward in the restricted pelvic space. An error in judgment may result in either part of the intramural ureter being left behind or inadvertent injury to the contralateral UO. In addition, the stapled margin cannot be assessed histologically. The Nagoya group reported stone formation at the staple line in 3 (5.7%) of 53 patients at an average of 20 months postoperatively [42]. Using a porcine model, Venkatesh et al. investigated the viability of bladder cells using 4 types of laparoscopic vascular and tissue stapler devices [43]. Viable cells were noted within the staple line in all cases and this could represent a potential risk for tumor recurrence in patients.

Romero et al. compared long-term safety and oncologic efficacy of extravesical laparoscopic stapling (*n* = 12) with the traditional transvesical open excision (*n* = 12) at nearly 4 years followup [44]. An increased positive margin rate (3 versus 0 patients) and local recurrence rate (2 versus 0 patients), and decreased recurrence-free interval were noted in the laparoscopic stapled group compared to the open group. However, none of these results was statistically significant probably owing to the small numbers. A number of authors have compared the various methods to distal ureteral excision and reported a higher incidence of positive surgical margins (up to 25%) and local recurrence (up to 15%) in the pure LNU with laparoscopic stapling cohort [25, 29, 45]. This highlights the need for meticulous removal of the whole ureter, UO, and bladder cuff. Contraindications include presence of mid/lower ureteral and bladder TCC.

More recently, Tsivian et al. described a variation on the laparoscopic stapling technique, using a 10-mm LigaSure Atlas (Valleylab, Tyco Healthcare UK Ltd, Gosport, UK) [46]. The bladder cuff was excised laparoscopically in an extravesical fashion using the LigaSure, without the need for staples. There were 2 bladder recurrences distant from the site of surgery but no reports of local recurrence in 13 patients followed up for nearly a year. Suturing of the bladder following LNU may be performed by those skilled in this technique.

### 4.2. Transvesical Laparoscopic Detachment and Ligation

This novel technique of securing the distal ureter and bladder cuff using transvesically placed laparoscopic ports was described by Gill et al. and is almost exclusively employed by the Cleveland clinic group [40, 41]. In this modified “pluck” procedure, a transurethral Collin's knife incision of the bladder cuff is performed after placement of a catheter into the affected ureter. Simultaneously, two 5-mm balloon-tipped ports are inserted suprapubically into the bladder. The incised UO is tightly snared using a 5-mm Endoloop (Ethicon, Cincinnati, Ohio, USA), preventing urine leakage from the ureter. Traction on the incised bladder cuff enables the mobilization of 3-4 cm of distal ureter into the bladder. The entire ureter can then be pulled through cephalad after radical nephrectomy and ureteral dissection. A bladder catheter is left in situ for 1 week.

The authors claim that the transvesical technique adheres to general oncological principles of complete and controlled en bloc specimen extraction. The ureteral catheter and Endoloop occlude the ureter, thereby reducing urine leakage. An indwelling ureteral catheter may aid identification and mobilization of the ureter during the laparoscopic procedure. Complete retrieval is confirmed by visualization of the Endoloop. However, this may be a difficult technique to master for most urologists and operating time is usually lengthened by 60–90 minutes [24, 47]. Other criticism of this approach includes the potential for irrigation fluid extravasation resulting in dilutional hyponatremia, the need for patient repositioning, and the possibility of port-site metastases. Contraindications include the presence of distal ureteral TCC or concomitant bladder tumors, prior pelvic surgery or irradiation and obesity [45].

LNU with the transvesical technique has been evaluated against ONU in a retrospective series [41]. Gill et al. reported that 27 patients who had undergone the former procedure had statistically significant superior results compared to the latter (35 patents) technique with regards to surgical time, blood loss, narcotic analgesia requirements, hospital stay, convalescence, and complication rates. Bladder recurrence rates and cancer specific survival were similar in both groups and no local retroperitoneal or port-site recurrence were diagnosed in any patients. The same group reviewed their outcomes in 60 patients following LNU, who had either had a laparoscopic stapling (*n* = 12) or transvesical laparoscopic detachment of the distal ureter (*n* = 36) [45]. Following a mean followup period of 23 months, positive margins were more common in the former group (25 versus 2.8%) as were the rates of bladder recurrences at the ipsilateral ureteral scar/orifice (41.7 versus 13.9%), retroperitoneal recurrence (8.3 versus 5.6%), and distant metastasis (25 versus 8.3%). None of these differences was statistically significant and definitive conclusions are difficult to derive from such small retrospective series.

Recently, Cheng et al. described a similar technique utilizing a pneumovesicum to secure the UO and bladder cuff [48]. Following initial cystoscopy to exclude bladder tumors, three 5-mm PediPorts (Tyco) are inserted suprapubically into the bladder. Following the establishment of a carbon dioxide pneumovesicum, the ipsilateral UO is closed using a stitch and the bladder cuff is incised down to fat using diathermy scissors. The bladder defect is closed using Polysorb sutures before completion of a standard 5-port laparoscopic nephroureterectomy. The authors report on a single case and propose that gas insufflation minimizes tumor spillage/seeding and permits a superior endoscopic view, even in the presence of bleeding, compared to liquid endoscopy as in the Cleveland method.

## 5. Open Removal

The open technique (either 2-incision or extended single incision) forms the standard against which all techniques are measured. Typically performed after nephrectomy, it can be performed through a lower midline, modified Pfannenstiel, or Gibson incision. The lower ureter is clipped, dissected free, and removed in continuity with the bladder cuff. The bladder cuff may be secured extravesically (using a right angle clamp) or via an anterior cystotomy. The en bloc specimen is delivered through the same incision.

In the review of 252 patients performed by Hall et al., 194 patients had undergone ONU with open bladder cuff excision for TCC and showed excellent long-term local control [6]. Klingler et al. reported outcomes at a mean followup period of 22 months in 19 patients who had an LNU with open bladder cuff excision versus 15 patients with a standard ONU [49]. Only 1 patient in the LNU group, with a high-grade, locally infiltrative, lymph node positive final histology (pT3b pN2 G3 TCC) had local recurrence. There was no significant difference in local recurrence rates between the laparoscopic and open group.

A recent multicenter retrospective Belgian study analyzed 100 patients following LNU for TCC [50]. Of these, 55 patients had an open excision of the distal ureter while the rest (45 patients) underwent a laparoscopic technique. Local recurrence was noted in 13 out of 100 cases of which 6 of 55 cases (11%) had open distal ureter management and 7 of 45 (16%) laparoscopic handling of the distal ureter. The investigators attribute the higher local recurrence rate to the larger proportion of high grade disease being operated on.

The open technique is not without its pitfalls. The “blind” extravesical clamping may compromise the contralateral UO and does not inevitably guarantee adequate bladder cuff retrieval [51]. An anterior cystotomy must be avoided in the presence of active bladder TCC as it retains the potential to seed tumor into the extravesical space. Furthermore early ligation/clipping of the ureter during the nephrectomy part is advisable. Additionally, prior pelvic surgery or irradiation and obesity may render the open procedure more challenging. Notwithstanding these potential concerns, the open approach to distal ureteral removal is oncologically sound and minimizes tumor spillage and therefore has withstood the test of time [52]. An open incision is often required for specimen extraction and adds little to overall morbidity, while providing visual confirmation of complete upper tract resection. It also enables accurate histological examination and reporting by the pathologist. Patient repositioning is usually required but not always mandatory. At our institution, this is the preferred method of dealing with the distal ureter following LNU. Table 1 summarizes the data from some of the studies (all retrospective in design) comparing the different techniques of distal ureteral management and associated outcomes.

## 6. Open or Laparoscopic?

Given the uncommon nature of upper tract TCC, there is a scarcity of prospective randomized studies with long-term followup comparing both modalities of nephroureterectomy. The “gold standard” open surgery offers excellent access but at the expense of increased patient morbidity. Since being first described in 1991 by Clayman et al., increasing surgical experience and equipment quality has seen LNU emerge as a viable option with the express intention of minimizing surgical morbidity without compromising oncology outcomes.

Rassweiler et al. performed a meta-analysis comparing ONU and LNU, which included 1365 patients from 85 studies [55]. LNU was associated with a slightly longer operating time (276.6 versus 220.1 minutes); significantly lower blood loss (240.9 versus 462.9 mL); decreased analgesia requirements and shorter hospital stay (not statistically significant in all included studies). There appeared to be no significant difference in complication rates, both minor (12.9 versus 14.1%) and major (5.6 versus 8.3%) between LNU and ONU, respectively. In addition, bladder recurrence, local recurrence, distant metastases, and actual disease-free two-year survival rates (75.2 versus 76.2%) were similar. It is worth noting that caution is suggested in interpreting the data from this meta-analysis as the majority of studies were retrospective, nonrandomized, and limited by short followup periods and variable outcome measures.

Further, recent smaller retrospective comparisons by McNeill et al. (*n* = 67) [56], Tsujihata et al. (*n* = 49) [53], Taweemonkongsap et al. (*n* = 60) [54], Rouprêt et al. (*n* = 46) [57], and Manabe et al. (*n* = 224) [58] have demonstrated parity between ONU and LNU when comparing oncological parameters over a shorter followup period (1–3 years). In a similar study, but with 7-year outcome data, LNU was noted to have a similar local recurrence rate (8 versus 15.4%), bladder recurrence rate (28 versus 42%), and 5-year metastases free survival rate (87.2 and 82.1%) compared to ONU [59].

Port-site seeding following LNU remains a concern but is fortunately rare if appropriate surgical techniques are adopted and have been mainly confined to individual case reports [60, 61].

Hand-assisted laparoscopic nephroureterectomy referred to as (HALNU) is often seen as a compromise between the open and laparoscopic technique. Arguments in its favor include a shorter learning curve, facilitates tactile feedback and the eventual requirement of a longer incision following LNU for specimen extraction. In a prospective, but nonrandomized study with 27 subjects, patients undergoing an HALNU could expect a quicker discharge from hospital, faster recovery, and fewer complications with an equivalent intermediate-term oncologic outcome compared to the open approach [62]. On the other hand, HALNU took longer and was more expensive. Other studies evaluating ONU and HALNU have confirmed an overall equivalence with regards to cancer control in the short term [30, 63].

Clearly, in the absence of prospective, randomized studies comparing ONU, LNU, and HALNU, it is injudicious to draw robust conclusions regarding the superiority of one technique over the other. Nevertheless, though radical ONU still represents the gold standard for upper tract TCC, laparoscopic nephroureterectomy appears to offer the advantages of minimally invasive surgery without deteriorating the oncological outcome in most cases.

## 7. Conclusion

TCC of the upper urinary tract, though rare, constitutes a serious urological disease. Even though it is curable in its early stages, there has been little improvement in disease-specific survival in high-risk patients over the last three decades. Radical nephroureterectomy, with en bloc removal of the entire ureteral length and cuff of bladder, remains the procedure of choice, and the technique has undergone numerous modifications in recent years. The integration of laparoscopy into urological practice has seen LNU emerge as a viable option for the management of upper tract TCC.

Given the relative rarity of the disease and a lack of robust multicenter effort, it is unlikely that the issue of LNU versus ONU for upper tract TCC will be resolved in a prospective randomized manner. Nevertheless, a multitude of small prospective studies with intermediate followup have clearly demonstrated the benefits of minimally invasive surgery (lesser morbidity, quicker recovery, better cosmesis) associated with LNU, along with comparable oncological efficacy in the hands of appropriately trained and experienced laparoscopic urologists. Long-term data from these studies would be beneficial.

The issue of the distal ureteral remains unresolved, and number and complexity of available techniques will undoubtedly continue to evolve. The existing data does not confirm the overwhelming superiority of one technique over the other. Each method has its distinct advantages and disadvantages and it is essential that the responsible surgeon adopts a meticulous, safe, reproducible, and oncologically sound technique. 

## Figures and Tables

**Table 1:** Summary of perioperative results and oncological outcomes for the different distal ureteral management techniques from selected larger studies. (ND: no data; LOS: length of stay; f/u: mean followup).

	f/u	*n*	Procedure type	Number of complications of distal ureter technique	Median LOS (days)	Positive margins	Bladder recurrence (%)	Pelvic recurrence (no)	No. of cancer-specific deaths
*Pluck technique*									
Palou et al., 1995 [13]	20	31	ONU	1	ND	ND	35	0	2
Ubrig et al., 2004 [15]	44	18	ONU	1	11	ND	50	0	5
Geavlete et al., 2007 [16]	44	72	ONU	2	10	ND	24	0	9
Laguna and de la Rosette, 2001 (meta-analysis) [18]	n/a	129	ONU/LNU	3	ND	ND	24	4	ND
Ko et al., 2007 [23]	22	19	ONU/LNU	0	7.3	0	26	1	1

*Modification of Pluck technique*									
Agarwal et al., 2008 [26]	15	13	LNU	0	7.3	0	38	0	1

*Ureteral deroofing*									
Shalhav et al., 2000 [29]	24	24	LNU	2	6.1	ND	23	3	ND

*Pluck in HALNU*									
Wong and Leveillee, 2002 [32]	8	14	HALNU	ND	2	ND	14	ND	ND

*Laparoscopic extravesical ureteral stapling*									
Jarrett et al., 2001 [39]	24	25	LNU	0	4	1	48	0	2

*Laparoscopic transvesical ureteral ligation*									
Gill et al., 2000 [41]	11	42	LNU	1	2.3	3	24	0	2

*Open distal ureteral excision*									
Klingler et al., 2003 [49]	22	19	LNU	0	8.1	0	ND	0	ND
Tsujihata et al., 2006 [53]	ND	49	ONU/LNU	0	4	ND	31	0	2
Taweemonkongsap et al., 2008 [54]	27	60	ONU/LNU	0	9	ND	37	3	8
